# Reliable reference genes and abiotic stress marker genes in *Klebsormidium nitens*

**DOI:** 10.1038/s41598-022-23783-9

**Published:** 2022-11-08

**Authors:** Pauline Chatelain, Cécile Blanchard, Jeremy Astier, Agnès Klinguer, David Wendehenne, Sylvain Jeandroz, Claire Rosnoblet

**Affiliations:** Agroécologie, CNRS, INRAE, Institut Agro, Université de Bourgogne, Université Bourgogne Franche-Comté, Dijon, France

**Keywords:** Biological techniques, Molecular biology, Plant sciences

## Abstract

Microalgae have recently emerged as a key research topic, especially as biological models. Among them, the green alga *Klebsormidium nitens,* thanks to its particular adaptation to environmental stresses, represents an interesting photosynthetic eukaryote for studying the transition stages leading to the colonization of terrestrial life. The tolerance to different stresses is manifested by changes in gene expression, which can be monitored by quantifying the amounts of transcripts by RT-qPCR. The identification of optimal reference genes for experiment normalization was therefore necessary. In this study, using four statistical algorithms followed by the RankAggreg package, we determined the best reference gene pairs suitable for normalizing RT-qPCR data in *K. nitens* in response to three abiotic stresses: high salinity, PEG-induced dehydration and heat shock. Based on these reference genes, we were able to identify marker genes in response to the three abiotic stresses in *K. nitens*.

## Introduction

Investigations on microalgae recently appeared as an emerging research topic due to their use in food industry, biofuels and health-related products^1–3^. For decades, several microalgae including *Micrasterias denticulata*^4,5^, *Closterium ehrenbergii*^6,7^ or *Chlamydomonas reinhardtii*^8,9^, have been the subject of fundamental research addressing the central question of the physiology and the evolution of photosynthetic eukaryotes. More recently, *Klebsormidium nitens*, a filamentous charophyte green algae, has been described as a new model for investigating the transitional steps leading to terrestrial colonization by aquatic algae^10^. Indeed, *K. nitens* displays increased resistance to specific stresses of terrestrial life such as desiccation or temperature fluctuations^11–13^. In addition, *K. nitens* possesses primitive cellular pathways related to hormones signaling^10,14^, as well as protein domains of arbuscular mycorrhiza-associated receptors typical of vascularized plants^15^. *K. nitens* has been also identified as a new potential candidate for biotechnological uses and commercial production of microalgal oils^16,17^.

Physiological responses promoting stress tolerance are manifested through adaptive changes in gene expression and modifications of transcripts stability. In this context, transcriptomic approach by RT-qPCR is a powerful tool to quantify transcript amounts and is frequently used to analyze fine-tuning gene expression^18–20^ thanks to its high sensitivity, specificity, accuracy, and reproducibility^21^. Obtaining reliable RT-qPCR data requires the normalization of the results using reference genes whose mRNA amount should remain unchanged under the experimental conditions of interest^22–24^. A selection of suitable reference genes have already been made for various algae under different stress conditions such as *Closterium ehrenbergii*^25^, *Chlamydomonas* sp. ICE-L^26^, *Ulva linza*^22^ or *Chorella pyrenoidosa*^27^. However, despite the growing interest for *K. nitens*^14,15,28–30^ in the scientific community, no studies have been dedicated to the search of reference genes in this algal species. Nevertheless, the identification of best reference genes for normalization of RT-qPCR data is definitively required for further analyses.

For this purpose, we determined the best couples of reference genes in *K. nitens* in response to three abiotic stresses: a high salt content, PEG-induced dehydration and heat shock. We selected 12 candidate genes previously cited as reference genes in algae^22,25,31^ or in other plant organisms^32,33^ and analyzed their expression stability in response to the abiotic stresses using four statistical algorithms: NormFinder^34^, GeNorm^35^, the ∆Cq calculation method^36^, and BestKeeper^37^. RankAggreg provided a ranked list of reference genes from most to least suitable^38^. Thanks to the selection of optimal reference genes, we then identified marker genes in response to the three abiotic stresses in *K. nitens*. The methodology for selecting reference genes was validated by studying the mRNA accumulation of one of these marker genes, the ATP-binding cassette transporter (*ABC*) encoding gene, in response to salt stress.

## Materials and methods

### Algal culture and stress treatments

*Klebsormidium nitens* strain NIES 2285 was cultured in liquid C medium^39^. Cultures were maintained at 20 °C, under stirring (130 rpm), 16 h photoperiod at 60 µmol m^−2^ s^−1^. *K. nitens* cells were harvested by vacuum filtration on cellulose ester membrane of 0.2 µm pores.

All stress treatments were applied after 2 weeks of cell culture. Cells were transiently exposed to heat shock (10 min, 45 °C) and were returned to control temperature (20 °C). Sampling was carried out during heat shock (0, 5 and 10 min) and 30 min after recovery from heat shock. Salt stress was applied at 500 mM NaCl (S0520.5000, 99.0% purity, Duchefa, Haarlem, Netherlands) during 1, 3 and 6 h or equal volume of C medium as control. PEG-induced dehydration was performed by adding PEG 6000 (8.07491.1000, Merck KGaA, Darmstadt, Germany) at 15% (w/v) and 30% (w/v) with time points of 1, 3 and 6 days. Unstressed algae, collected in parallel, were used as control. All harvested cells were immediately frozen in liquid nitrogen and stored at − 80 °C until further RNA extraction.

### RNA extraction, cDNA synthesis and RT-qPCR

Frozen cells were ground to a fine powder in liquid nitrogen using a MM200 oscillating vibro-mill (Retsch, Haan, Germany) and 5 mm diameter glass beads. Total RNA was isolated according to a protocol from Oñate-Sánchez and Vicente-Carbajosa^40^. DNA-free™ DNA Removal Kit (AM1906, Invitrogen, Thermo Fisher Scientific, Waltham, Massachusetts, USA) was used to remove gDNA contamination. RNA purity and quality were determined using a NanoDrop ND-1000, then RNA integrity was checked through electrophoresis on a 1% (w/v) agarose gel. First-strand cDNA was synthesized from 1 µg using RNA High-Capacity cDNA Reverse Transcription Kits (4368813, Thermo Fisher Scientific, Waltham, Massachusetts, USA) according to the manufacturer’s instructions. RT-qPCR were performed using GoTaq qPCR Master Mix kit (A6002, Promega, Madison, Wisconsin, USA) and the ViiA 7 Real-Time PCR System (Applied Biosystems, Thermo Fisher Scientific, Waltham, Massachusetts, USA). Reactions were performed in a final volume of 5 μL including 2.5 μL of GoTaq^®^ qPCR Master Mix 2x, 10 ng of cDNA and 0.25 μM of forward and reverse primers. PCR amplification was performed according to the following program: a first denaturation step (95 °C, 2 min) then 40 cycles, each consisting of a denaturation step (95 °C, 15 s), a hybridization step (65 °C, 30 s) and an elongation step (72 °C, 30 s).

### Selection of candidate reference genes

Twelve candidate genes were selected from literature and algae homologous sequences were then identified using BLASTn searches within the *Klebsormidium nitens* NIES-2285 genome project database v1.1 (http://www.plantmorphogenesis.bio.titech.ac.jp/~algae_genome_project/klebsormidium/), including the genome and the transcriptome of *K. nitens* (Table 1). Sequence with at least 70% identity between the species of origins and *K. nitens* were selected. Primers sequences were designed using Primer3Plus (http://www.bioinformatics.nl/cgi-bin/primer3plus/primer3plus.cgi) (Table 1). Primer specificity was assessed via RT-qPCR, melting curve analysis (− dFluorescence/dTemperature) and 2% (w/v) agarose (> 99.8%, Eurobio Scientific, Courtaboeuf, France) gel electrophoresis of the qPCR products (Supplementary Fig. S1). The PCR amplification efficiency (E) for each primer pair were calculated using the LinRegPCR software^41^.

**Table 1:** Candidate reference genes and associated primers set used in RT-qPCR.

Gene name	Gene symbol	Gene ID	Gene ID Homolog and species	Primer sequence (5′–3′) forward/reverse	Size (bp)	E	References
Eukaryotic translation initiation factor 3G	*eIF3G*	kfl00006_0710	JN885965.1 *Ammopiptanthus mongolicus*	AAGCACGGCAGGTTGTAAGT/AAGGATGAAACGCACCGACA	141	1.899	Shi et al.^32^
Eukaryotic translation initiation factor eIF-6	*eIF6*	kfl00065_0190	KM229561.1 *Closterium ehrenbergii*	ACATCTTGGTCGGCAGCTAC/CGACCAGGGGGACTTGTAAC	114	1.759	Lee et al.^25^
Actin and related proteins	*ACT*	kfl00726_0080	*AK100267.1* *Oryza sativa L*	AAGCTTGCATATGTGGCCCT/ATGACTTGGCCGTCAGGAAG	101	1.903	Moraes et al.^33^
Ubiquitin family protein	*UBQ*	kfl00037_0290	AK101547.1 *O. sativa* L.	TCCTCACAAGCAGCGTCAAA/GCTGTTGGAACTTGAAGGCG	100	1.890	Moraes et al.^33^
Ubiquitin/40S ribosomal protein S27a fusion	*Ub/S27*	kfl00144_0120	AK061988.1 *O. sativa L*	AGTGCGGGTTGACCTATGTG/TGTGTGTCGAGCTGAAGCAA	136	1.891	Moraes et al.^33^
40S ribosomal protein S26	*S26*	kfl00199_0140	XM_001691849.2 *Chlamydomonas reinhardtii*	GCCTCCACAGTTGCACATTG/CCGTCTGTAAAGTCCGGCAT	112	1.893	Simon et al.^31^
Mitochondrial or chloroplast ribosomal protein L34 precursor	*L34*	kfl00870_0030	XM_001697888.2 *C. reinhardtii*	GGGTTTTGGCAAGGCGAATC/GCAAGGTAGTTCCCCACTCC	111	1.887	Simon et al.^31^
Histone H4	*H4*	kfl00394_0030	KM229560.1 *C. ehrenbergii*	AGAATCTACCAGCTCCCCGT/CTTGGTGATGCCCTGGATGT	138	1.867	Lee et al.^25^
Histones H3 and H4	*H3*	kfl00509_0020	JN543390.1 *Ulva linza*	AAGCCTCATCGTTATCGCCC/CAATTTCCCTGACAAGGCGC	115	1.885	Dong et al.^22^
Beta tubulin	*TUB1*	kfl00215_0170	JN543388.1 *U. linza*	CGACCCCACTGGAACATACC/CCAGATCCATGAGCACAGCA	116	1.865	Dong et al.^22^
Alpha tubulin	*TUB2*	kfl00203_0030	JN543389.1 *U. linza*	TGCTGAGAAGGCTTACCACG/ACGCCATGTACTTACCGTGG	113	1.900	Dong et al.^22^
Citrate synthase	*CitS*	kfl00009_0420	–	CAGAAACACGTTCAAGGGCG/GTGGATGGTGAGGTACAGCC	133	1.896	Ohtaka et al.^14^

*K. nitens* genes ID, the homologous genes and the related publications are referenced. Amplicon size and RT-qPCR efficiency (E) are indicated.

### Analysis of gene expression stability and ranking of candidate reference genes

The stability of the candidate genes was evaluated in silico using four algorithms, GeNorm^35^, NormFinder^34^, BestKeeper^37^, and the ∆Cq method^36^. GeNorm calculation, as Normfinder, are based on fold change values, whereas BestKeeper and the ∆Cq methods are based on raw Cq values. GeNorm calculates the average pairwise variation of gene expression of each candidate compared to the others. A stability value (M) is then assigned to each gene. This M value must be lower than the threshold of 1.5 set by the software developers. The most stable reference gene has the lowest M value. NormFinder calculates the stability value (SV) based on the overall expression variation of candidate genes for normalization, and also the variation between subgroups of samples. The BestKeeper algorithm calculates the stability of candidate reference genes based on the coefficient of variance and standard deviation of gene expression (CV). The reference gene with the lowest CV is considered as the most stable gene. The ΔCq method consists of comparing the relative expression of each pair of genes. All comparisons for one gene allowed calculation of mean standard deviation (mSD) that was used to assess the stability of the gene expression of the 12 candidates: low mSD value indicated a stable expression. Therefore, a final ranking was determined using the R RankAggreg package which combines the different rankings by aggregation via a Cross-Entropy algorithm^38^.

### Determination of stress marker genes

Several stress-related genes were selected based on their homologous function described in the literature in *Arabidopsis thaliana*. Algae homologous genes were identified by BLASTn method with the *Klebsormidium nitens* NIES-2285 genome project database v1.1 including the genome and the transcriptome of *K. nitens* (Supplementary Table S1). Relative gene expressions were analyzed using the common base method from Ganger^42^. Statistical analyses were performed by Kruskal–Wallis test followed by Dunn test and p-value were adjusted by Holm method, between treated and control samples for salt and PEG stress, and between each point for heat shock.

## Results

### Selection of candidate reference genes

The aim of this work was to identify genes suitable as reference genes in *K. nitens,* showing the most stable expression in response to abiotic stress. The characteristic and primer sequences of the 12 candidate reference genes selected through a bibliographic review are presented in Table 1.

**Figure 1:**
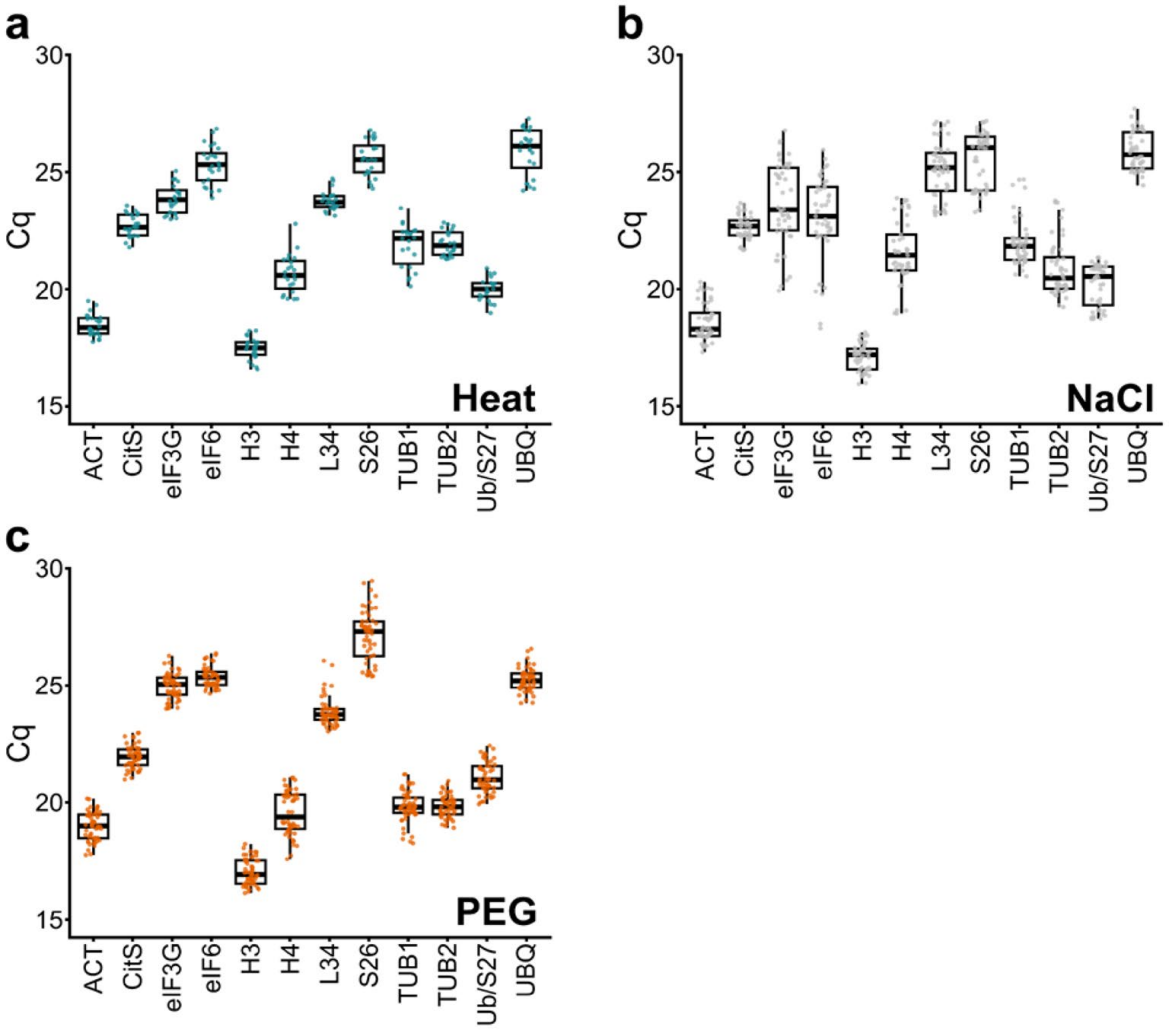
Quantification cycle (Cq) variation of candidate reference genes in each stress condition. Each value of Cq from control and stress samples is represented by a dot. Box indicates the 1st and 3rd quartiles, the ends of vertical line represent the maximum and minimum values. The horizontal line through the box represents the median. Cq from control and stressed samples after: (**a**) 5 and 10 of heat shock at 45 °C, then 30 min at 20 °C, (**b**) salt stress with 500 mM NaCl during 1, 3 and 6 h and (**c**) PEG-induced dehydration at 15 and 30% (w/v) during 1, 3 and 6 days.

In order to study the stability of their expression during abiotic stress conditions, we first analyzed the amounts of the corresponding mRNAs at basal state and in response to heat shock, high concentration of salt and dehydration, following the quantification cycle (Cq) value. The Cq value reflected the amount of transcripts (a lower Cq correlated with a higher level of mRNA) and the height of each box and vertical line displayed the variability of the mRNA amounts of control and stressed samples (Fig. 1). Following the three treatments, the Cq values of the potential reference genes ranged from 15.95 to 29.46, with 90% ranging from 18.00 to 26.00 (Fig. 1, Supplementary Dataset 1, 2). Among all the samples, the least abundant transcripts were those encoding eIF6, S26, and UBQ (mean Cq values over than 25.00 in response to the three stresses), while H3 emerged as the most abundant transcripts (mean Cq values of 17.03, 17.09 and 17.47 in response to PEG, salt, and heat stress, respectively; Fig. 1a–c). Concerning the variability of the control and treated sample transcript levels, in response to heat shock L34 showed the lowest variability with Cq between 23.14 and 24.73, while UBQ mRNA content is the most affected (values ranging from 24.20 to 27.28; Fig. 1a). Regarding salt stress, the lowest variability was found for CitS mRNA, while that of eIF6 is the most variable (Fig. 1b; Supplementary Dataset 2). On the contrary, under PEG stress, eIF6 mRNA displayed the lower dispersal of the Cq value and S26 mRNA the highest one (Fig. 1c; Supplementary Dataset 2). These Cq analyzes highlighted the need to identify reference genes adapted to each stress conditions. To obtain a more robust evaluation of candidates, we performed statistical tests to rank genes using four algorithms: NormFinder^34^, GeNorm^35^, the ΔCq calculation^36^ (dCq), and BestKeeper^37^.

### Expression stability of candidate reference genes

**Figure 2:**
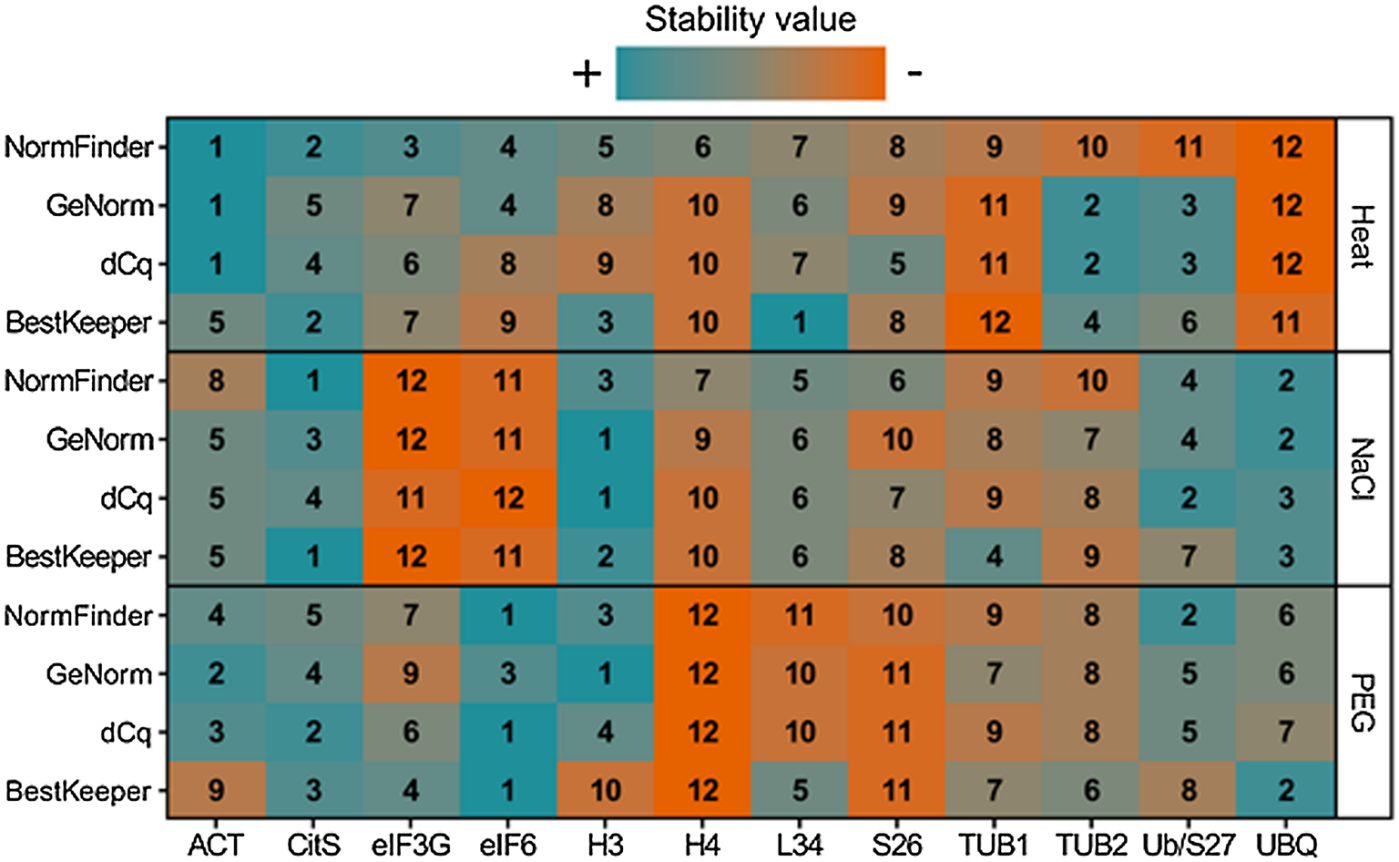
Expression stability and ranking of candidate reference genes for each algorithm, NormFinder, GeNorm, dCq, BestKeeper and in response to heat shock 10 min at 45 °C then 20 °C, salt stress at 500 mM NaCl and 15 and 30% (w/v) PEG-induced dehydration stress. The numbers corresponding to the rank of each candidate were calculated from the SV, M, mSD, CV values for NormFinder, GeNorm, dCq and BestKeeper, respectively.

Several statistical algorithms have been developed to select stably expressed reference genes for accurate normalization of RT-qPCR data. We have chosen four algorithms commonly used in reference gene determination: NormFinder^34^, GeNorm^35^, dCq^36^, and BestKeeper^37^. Each method led to the ranking of reference genes based on their stability during the conditions tested: from the most (rank 1) to the least (rank 12) stable, calculated from SV (NormFinder), M (GeNorm), mSD (dCq) values and CV (BestKeeper). Each algorithm focused on different criteria to determine stability. Actually, GeNorm did not consider variation in mRNA amounts within samples, while Bestkeeper eliminated candidates with a standard deviation of Cq greater than 1. NormFinder is the only one that considered variations in transcript levels within treatment. The dCq method focused only on the standard deviation of the pairwise difference between the Cq of each candidate gene. The information provided by these four methods were complementary. In order to limit biases in the selection of the optimal reference genes in our conditions, the rankings obtained from each algorithm were separately evaluated and summarized in Fig. 2.

Although the classification criteria were different, the rankings obtained with each algorithm shared similarities within each treatment (Fig. 2). For example, *ACT* was ranked 1 by NormFinder, GeNorm and dCq, and 5 by BestKeeper in response to heat stress, while *UBQ* were ranked 12 by NormFinder, GeNorm and dCq, and 11 by BestKeeper. Following salt stress, *H3* was ranked 3 by NormFinder, 1 by GeNorm and dCq, and 2 by BestKeeper while *eIF3G* was ranked 12 by NormFinder, GeNorm and BestKeeper, and 11 by dCq. Regarding PEG treatment, *eIF6* appeared to rank 1 in the rankings of NormFinder, dCq and BestKeeper, and to rank 3 in the ranking of GeNorm, whereas *H4* was ranked 12 by each algorithm (Fig. 2). The rankings described above highlighted the difference in stability of the transcripts and therefore the importance of choosing reference genes which expression was independent of the experimental conditions. While the rank of some candidate genes was similar whatever the algorithm, for others it differed. For instance, discrepancy appeared for *L34*. It was found to be the most stable gene by BestKeeper under heat stress conditions, while it was only ranked 7, 6, and 7 by NormFinder, GeNorm and dCq, respectively. Also, *H3* was reported as stable under PEG treatment by GeNorm (rank 1), NormFinder (rank 3), and dCq (rank 4), while it emerged as unstable with BestKeeper at rank 10 (Fig. 2).

**Figure 3:**
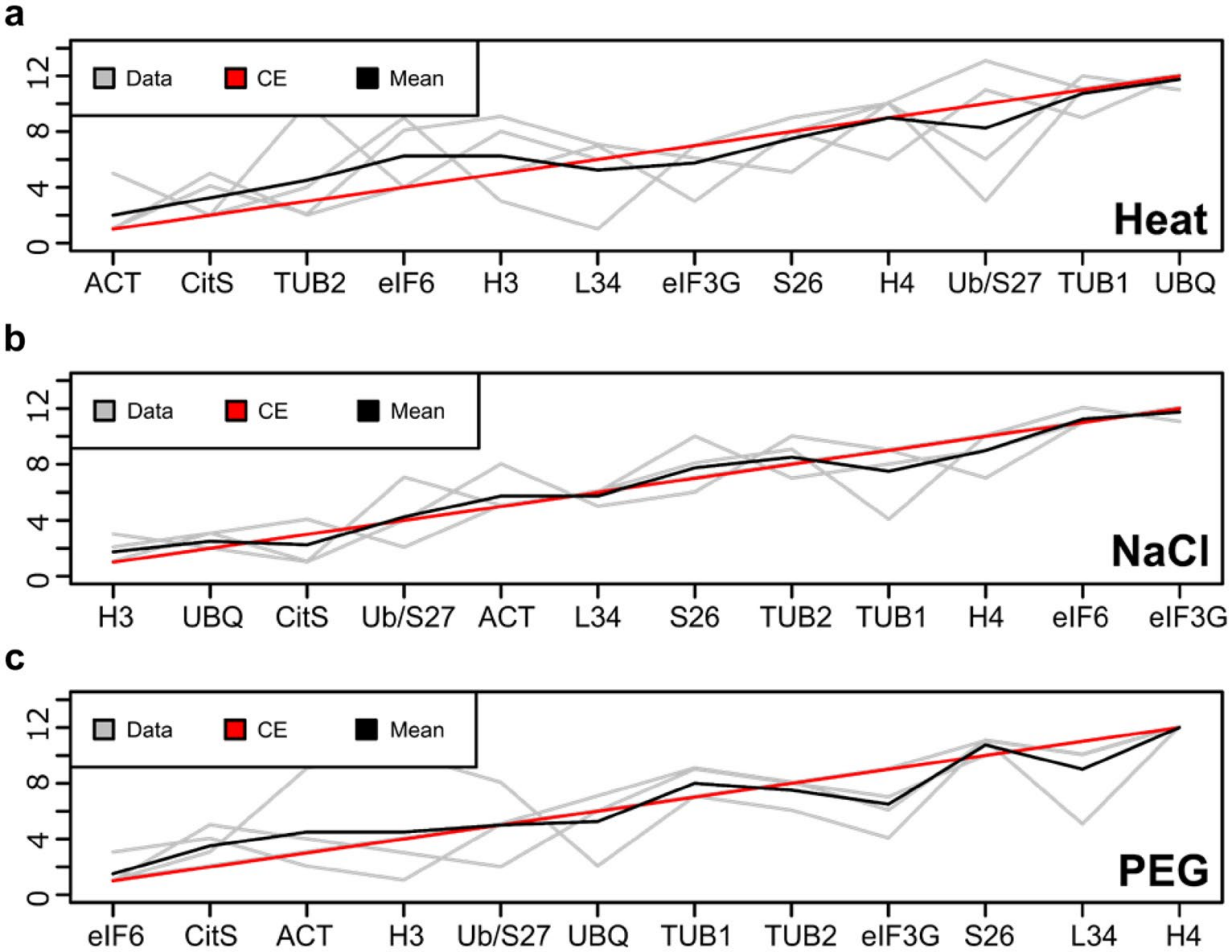
Rank aggregation of candidate reference genes for each algorithm in the three stress conditions, (**a**) heat shock, (**b**) salt stress and (**c**) PEG-induced dehydration. Analysis was performed by RankAggreg package in R software. Grey lines corresponding to ranking by NormFinder, GeNorm, dCq and BestKeeper, black line represents the mean rank of each methods and red line the results of best rank combination determined by the Cross-Entropy algorithm (CE).

As mentioned above, each method of calculation was complementary and, in order to combine these different rankings together and to overcome any contradictions between some algorithms, the R package RankAggreg^38^ was used. This method allowed us to reorganize the ranks independently of the origin of the data^38^, thus making it possible to compare the results from the four algorithms. RankAggreg led to the determination of an optimal ranking list in each stress condition (Fig. 3). Thus, in case of heat stress, the most stable expressed gene was *ACT* (Fig. 3a), the best one for salt stress was *H3* (Fig. 3b), while *eIF6* displayed the most stability following PEG-induced dehydration (Fig. 3c). Since the use of multiple reference genes allows optimal results^21^, we decided to consider two reference genes per condition. Finally, *ACT* and *CitS*, *H3* and *UBQ*, and *eIF6* together with *CitS*, were selected as couples of reference genes for RT-qPCR normalization for studies in *K. nitens* species, in conditions of heat shock, salt stress and PEG-induced dehydration stress, respectively (Fig. 3).

### Selection of abiotic stress marker genes

Once suitable reference genes selected, we searched for marker genes of the different abiotic stresses. For this purpose, 26 candidate genes exhibiting variations under abiotic stress conditions in *Arabidopsis thaliana* or in a recent RNAseq experiment conducted in *K. nitens*^28^ were selected (Table S1). The dynamic of expression for these genes in response to heat shock, salt stress and PEG-induced dehydration, were monitored by RT-qPCR.

**Figure 4:**
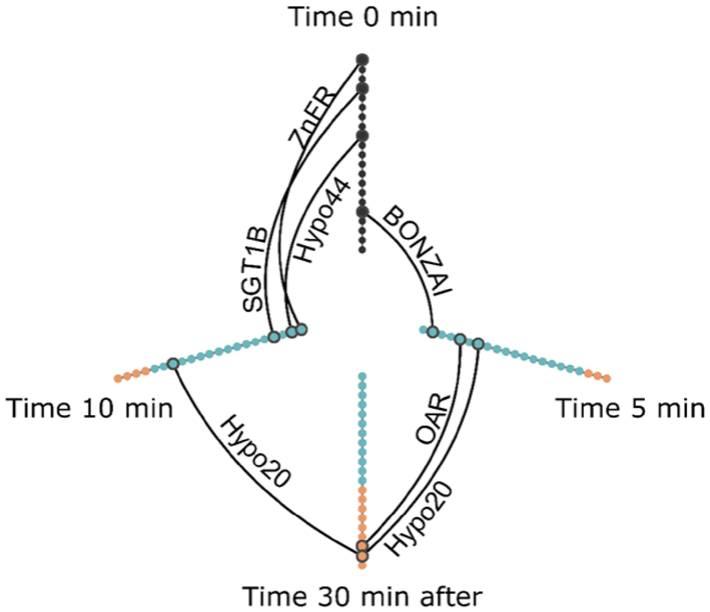
Hive plot depiction of statistical differences of candidate gene marker mRNA abundances depending on the heat shock treatment time. Each axis represents a time point during the heat shock at 45 °C: 0 min, 5 min, 10 min then 20 °C for 30 min. Each candidate marker gene is represented by a node and are ordered alphabetically on the top axis. On the other axis nodes are ordered according to their fold change, i.e., their transcript level under heat shock stress conditions compared to the one at time 0. On theses axes, nodes represented in blue presented a fold change lower than 1 and those in orange presented a fold change upper than 1. Edges connecting correspond to a statistical difference between the fold change of the two time points for one gene (Kruskal–Wallis followed by Dunn test, P < 0.05, n = 4).

In case of heat stress, we represented the transcript accumulation at each time point (0 min, 5 min, 10 min + 30 min) relative to time point 0 on a hive plot (Fig. 4). Each node corresponded to a gene and was colored according to it fold change. Genes whose amounts of transcripts significantly increased or decreased were linked between the two relevant time points. During heat shock, the majority of the genes exhibited a trend toward a decreased level of mRNA, illustrated by the prevalence of blue dots in axes of time points 5 min and 10 min (Fig. 4). One gene, *BONZAI*, coding for a calcium-dependent phospholipid-binding protein, displayed a statistically different decrease during the first 5 min at 45 °C. Likewise, mRNA level of three genes significantly decreased between time zero and 10 min: *ZnFR*, encoding a zinc finger containing protein, *SGT1B* for a chaperone binding protein and *Hypo44*, for a protein showing homology with a cell wall integrity and stress response component in *A. thaliana*. No significant increase in mRNA content was measured for the selected genes at the time point 30 min (Fig. 4). However, two genes encoding the oxidoreductases, *Hypo20* and *OAR*, were down-regulated during a 30 min recovery after heat shock (Fig. 4).

**Figure 5:**
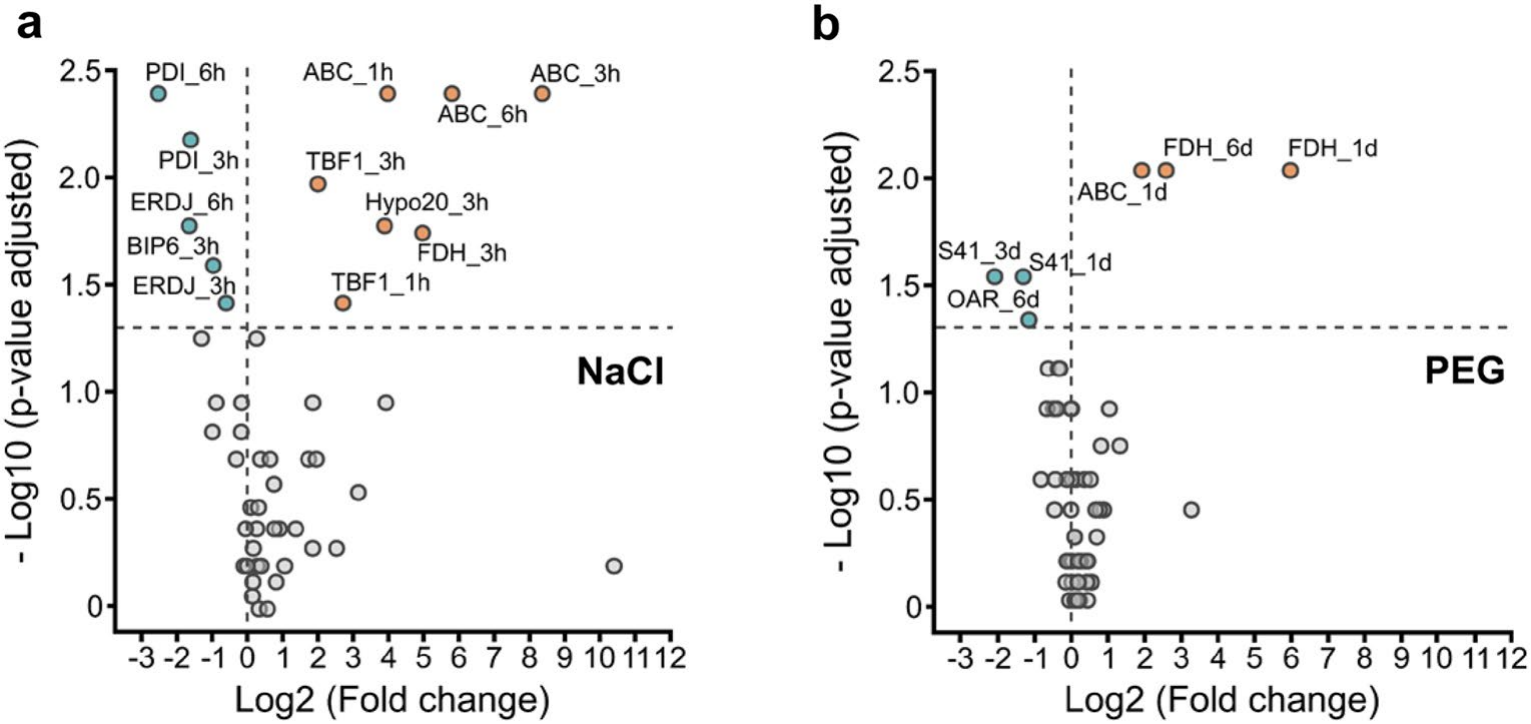
Volcano plot of mRNA level changes of candidate marker genes relative to control, in response to (**a**) 500 mM of NaCl (salt stress), (**b**) 30% (w/v) PEG-induced dehydration. Each dot represents one gene at one time point labelled: Name_Time point. Gene statistically up-regulated were labelled in orange (positive Log2) and in blue even down-regulated (negative Log2). Statistical differences between treated sample and control were determined using Kruskal–Wallis followed by Dunn test and p-values were adjusted with Holm method (*P < 0.05, n = 5).

Only seven genes showed an amount of transcripts significantly affected under salt stress (Fig. 5a). Four of them were up-regulated: *TBF1*, coding for a DNA-binding transcription factor, *ABC*, *Hypo20* and *FDH* that codes for a mitochondrial formate dehydrogenase while three were down-regulated: *PDI* (Protein Disulfide Isomerase), *ERDJ* and *BIP6*, both coding for heat shock proteins. Hypo20, FDH, BIP6 transcripts accumulation was transiently modulated whereas ABC, TBF1, PDI, ERDJ transcripts showed continuous variations during treatments. Analysis of candidate gene expressions in response to PEG-induced dehydration revealed four genes with statistically different level of transcripts compared to control (Fig. 5b). Indeed, *ABC* and *FDH* appeared to be up-regulated, while *S41*, that codes for a peptidase, and *OAR* genes were down-regulated (Fig. 5b). All genes identified with statistically different fold changes (Figs. 4, 5) could be considered as markers for abiotic stress in *K. nitens*.

**Figure 6:**
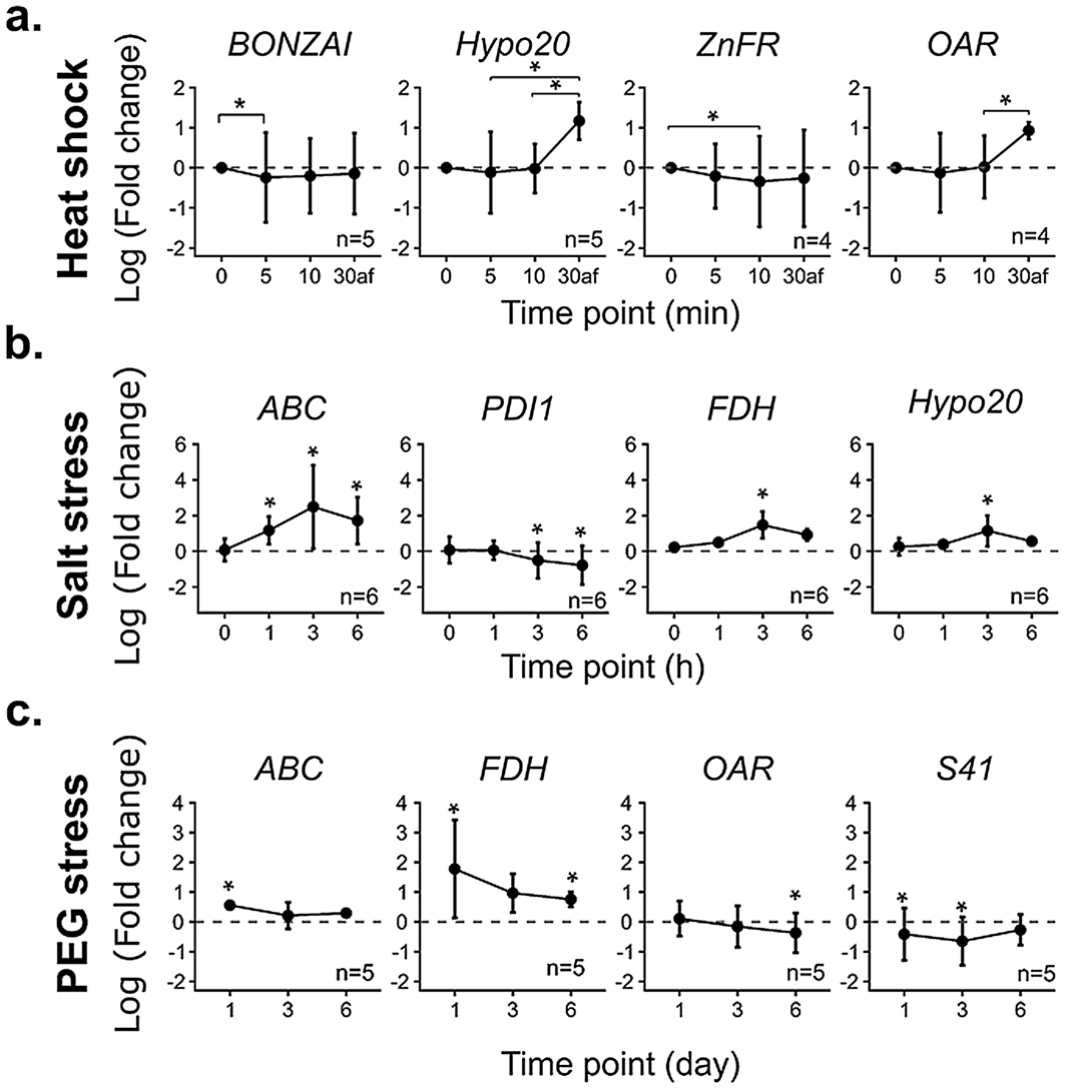
Log transform expression pattern of four marker genes in each abiotic stress normalized to the most adapted pair of reference genes. (**a**) BONZAI, Hypo20, ZnFR and OAR mRNA amounts relative to control at 0 min were measured in response to heat shock. 30af: 30 min at 20 °C after heat shock. Statistically significant differences between each time point were determined using Kruskal–Wallis followed by Dunn test (*P < 0.05). (**b**) ABC, PDI1, FDH and Hypo20 transcripts accumulation relative to control were calculated in response to salt stress. Statistically significant differences between treated sample and control were determined using Kruskal–Wallis followed by Dunn test (*P < 0.05). (**c**) ABC, FDH, OAR, and S41 mRNA amounts relative to control were quantified in response to 30% (w/v) PEG induced dehydration stress. Statistically significant differences between treated sample and control were determined using Kruskal–Wallis followed by Dunn test (*P < 0.05).

The monitoring of the accumulation of transcripts from nine genes (Fig. 6) demonstrated various behaviors according to the gene and the applied stress. Overall, our results provided a useful set of marker genes for studying gene expression in *K. nitens* under different abiotic stress conditions.

### Validation of reference genes

**Figure 7:**
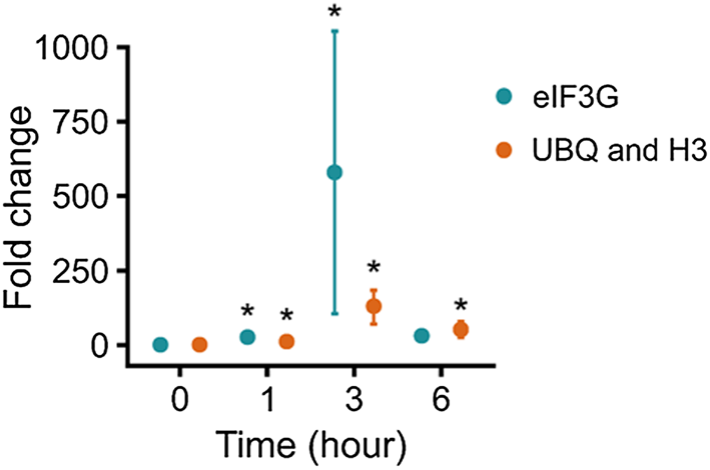
KnABC transcripts accumulation under salt stress condition relative to control. Each dot represents the mean of six biological repetitions, error bars represent standard errors of the mean. The quantification was normalized with the worst reference gene candidate eIF3G in blue or with the optimal pair of reference genes UBQ and H3 in orange. Statistically significant differences between NaCl treatment and control were determined using Kruskal–Wallis followed by Dunn test (*P < 0.01).

To demonstrate the reliability of the selected reference gene couples, we analyzed the expression level of the *ABC* gene in response to salt stress (Fig. 6b). In order to highlight the importance of adequate normalization, quantification of KnABC transcripts obtained by RT-qPCR under salt stress were normalized with the most appropriate reference genes pair (*H3* + *UBQ*) and with the most inappropriate reference gene (eIF3G; Fig. 7). With the normalization of RT-qPCR using *H3* and *UBQ*, KnABC mRNA amount statistically increased in the treated samples compared to the control as early as one hour of treatment with a fold change of 11. After three hours of treatment, a maximum fold change of 130 was reached, then half decreased after six hours, but remaining statistically different from the control (Fig. 7).

The normalization using *eIF3G* as reference gene resulted in differences that could distort interpretations. After 1 h, a statistically different increase was observed compared to control, whatever the reference genes used. After 3 h, the amount of KnABC mRNA reached its maximum fold change but the normalization with *eIF3G* led to a fourfold overestimation and considerable spread of standard errors compared to the result obtained with the most appropriate reference genes. Finally, normalization with *eIF3G*, instead of the one with *H3* and *UBQ*, resulted in the lack of a statistical difference in transcripts accumulation between NaCl-treated samples and controls after 6 h of treatment (Fig. 7). These results highlighted the need for suitable and optimized reference genes for normalization of RT-qPCR result.

## Discussion

Due to its importance as a model organism, studies using *K. nitens* have been increasingly published and have been reported investigations focusing, for instance, on the evolution of auxin^14^, jasmonate^28^, nitric oxide signaling^29^, the origin of mycorrhiza^15^, or the response to high light stress^30^. Transcriptomic approaches using RT-qPCR are commonly used, because of their simplicity and low cost for laboratories^21^. Therefore, one can predict that transcriptional analyses in *K. nitens* could expand in the coming years. Thus, the identification of adapted reference genes for the normalization step in RT-qPCR assay on *K. nitens* appeared necessary in order to obtain reliable results. Moreover, several studies have demonstrated the need of adapting reference genes to the experimental conditions^22–24^. In the present study, we explored the expression stability of 12 candidate reference genes in *K. nitens* in response to salt, heat, and PEG-induced dehydration stresses. Each of the candidate was previously described as a reference gene either in algae^22,25,31^ or in other plant organisms^32,33^ in response to different abiotic stresses.

The plot of Cq values gave first information on candidate variability expressions. However, the variability of Cq was not sufficient to determine optimal reference genes, since the stability of the expression but also an expression level above the background were required, according to the developers of Bestkeeper^37^.

The most suitable reference genes to normalize our results were selected using 4 algorithms. NormFinder provides a stability value for each reference gene by estimating the intra- and inter-group expression variation. GeNorm recommends selecting genes by pairwise variation between a gene and the average of the other. BestKeeper determines a ranking of gene stability by performing Pearson correlations. The dCq method ranks candidate genes solely based on their expression variability. Interestingly, the rankings obtained for each method and each stress differed, each algorithm using its own calculation method. Such differences led us to use the RankAggreg package, so the ranking lists obtained for each method could be combined into an optimal list of reference genes^38^.

Since it has been pointed out that RT-qPCR data normalized with a single reference gene present a bias^21,43,44^, we kept the first two genes as the most suitable reference gene pair for normalization of RT-qPCR data in *K. nitens* for each condition tested. This study highlighted *ACT* and *CitS*, *H3* and *UBQ*, and finally *eIF6* with *CitS*, in conditions of heat shock, salt stress and PEG-induced dehydration, respectively. These genes were already described as reference gene in other organisms. Indeed, *ACT* has been used for years as a reference gene, especially in Northern blot experiments^45^. Its use is recommended in rice in response to salt, or in common bean in response to various abiotic stresses^46^. Commonly used reference genes often display relatively high mRNA amounts in cells^45^, such as genes coding for histones. Accordingly, in *K. nitens,* H3 exhibited a low Cq in response to heat stress. Another criterion for the selection of reference genes is their involvement in basic cellular processes such as protein translation or ubiquitin-dependent protein degradation. For instance, *eIF6* and *UBQ* were used as reference genes in *A. thaliana*^47^ or in tomato leaves^48^.

Finding citrate synthase as a reference gene was less predictable, since up-regulation of citrate synthase has been observed in maize during germination and in response to light^49^, or in tea plants under drought stress^50^. However, citrate synthase has been selected as a reference gene in medicinal plant *Glycyrrihza*^51^ and in *K. nitens* in previous RT-qPCR experiments^14^.

In order to develop a full set of molecular tools for the analysis of *K. nitens* responses to environmental conditions, we investigated genes that were differentially expressed during heat shock, salt stress and PEG-induced dehydration. In response to heat shock, mRNA amounts of Hypo20 and OAR, two oxidoreductases, displayed a statistically significant increase. The induction of *OAR* (3-oxoacyl-acyl-carrier-protein reductase) that participates in lipid metabolism, appeared not so surprising since the composition of membranes is strongly impacted by temperature changes^52^. Furthermore, *OAR* has been previously detected as up-regulated in response to heat stress in the herbaceous peony *Paeonia lactiflora*^53^. Following heat shock, the level of ZnFR and BONZAI mRNA significantly decreased, whereas we expected an upregulation of these two genes. Indeed, BONZAI, a calcium-dependent phospholipid-binding Copine protein, has been previously described as being involved in the regulation of Ca^2^^+^ during osmotic stress^54^. In case of ZnFR, a Zinc finger RING-type domain containing protein, several proteins of the RING-protein family have been found to be up-regulated in response to heat stress, notably in hot pepper^55^ and in *A. thaliana*^56^. In *K. nitens*, *Hypo20* and *OAR* could be reliable indicators of the recovery after heat shock, while *ZnFR* and *BONZAI* could be used as early marker genes during heat shock.

During salt stress, *ABC*, *FDH* and *Hypo20* genes were up-regulated. As previously shown in rice and *A. thaliana*^57,58^, ATP-binding cassette transporter emerged as the most suitable salt stress marker gene. The mRNA level increase of FDH was also consistent with studies in various plants in response to both biotic and abiotic stresses^59^. During salt stress response in *K. nitens*, a Protein Disulfide Isomerase 1 (PDI1), involved in the ER stress response^60^, was down-regulated. Nevertheless, the gene expression of this type of enzyme is difficult to predict, since its expression variability is high even within the same species or between ecotypes, as in Chinese cabbage where no less than 32 PDI have been identified and forms a large multigene family^61^.

In the case of PEG-induced dehydration stress, *ABC* and *FDH* were also found to be up-regulated in *K. nitens*. As salt and heat shock also triggered osmotic stress, it is not surprising to find common marker genes between these stresses. For example, some ABCs genes have been found as up-regulated in response to NaCl and PEG stresses in *Brassica rapa*^62^. It was also interesting to observe that the decrease of the mRNA levels of OAR and S41 appeared at different time points during the PEG kinetics. *OAR* would thus indicate long-term dehydration stress while *S41* would be indicative of earliest responses.

Finally, to validate the relevance of our methodology for the reference gene selection in *K. nitens*, the KnABC transcript level was monitored under salt stress conditions and normalized with the most appropriate reference gene pair (*H3* + *UBQ*) and with the most inappropriate reference gene (*eIF3G*). Misinterpretations and overestimations have resulted from the use of *eIF3G* in monitoring KnABC transcript level. Previously, several cases of improper evaluation due to the use of inappropriate reference genes have already been reported^22,43,63,64^.

This study identified the molecular tools needed to produce reliable RT-qPCR data following abiotic stresses in the model algae *K. nitens*. Given the increasing amount of studies on this model, this work will facilitate further understanding of the biological adaptations that allowed the colonization of land by ancestral plant lineages.

## Supplementary Information

Supplementary Figure S1.

Supplementary Table S1.

Supplementary Information 1.

Supplementary Information 2.

## Data Availability

The data that support the findings of this study are available from the corresponding author upon reasonable request.

## References

[1] Wijffels RH, Barbosa MJ (2010). An outlook on microalgal biofuels. Science.

[2] Khan MI, Shin JH, Kim JD (2018). The promising future of microalgae: Current status, challenges, and optimization of a sustainable and renewable industry for biofuels, feed, and other products. Microb. Cell Factories.

[3] Barkia I, Saari N, Manning SR (2019). Microalgae for high-value products towards human health and nutrition. Mar. Drugs.

[4] Giddings TH, Brower DL, Staehelin LA (1980). Visualization of particle complexes in the plasma membrane of *Micrasterias denticulata* associated with the formation of cellulose fibrils in primary and secondary cell walls. J. Cell Biol..

[5] Steiner P (2021). Winter survival of the unicellular green alga *Micrasterias denticulata*: Insights from field monitoring and simulation experiments. Protoplasma.

[6] Lippert BE (1967). Sexual reproduction in *Closterium moniliferum* and *Closterium ehrenbergii*1. J. Phycol..

[7] Sathasivam R, Ebenezer V, Guo R, Ki J-S (2016). Physiological and biochemical responses of the freshwater green algae *Closterium ehrenbergii* to the common disinfectant chlorine. Ecotoxicol. Environ. Saf..

[8] Spalding MH, Portis AR (1985). A model of carbon dioxide assimilation in *Chlamydomonas reinhardii*. Planta.

[9] Fauser F (2022). Systematic characterization of gene function in the photosynthetic alga *Chlamydomonas reinhardtii*. Nat. Genet..

[10] Hori K (2014). Klebsormidium flaccidum genome reveals primary factors for plant terrestrial adaptation. Nat. Commun..

[11] Nagao M, Matsui K, Uemura M (2008). *Klebsormidium flaccidum*, a charophycean green alga, exhibits cold acclimation that is closely associated with compatible solute accumulation and ultrastructural changes. Plant Cell Environ..

[12] Holzinger A (2014). Transcriptomics of desiccation tolerance in the streptophyte green alga Klebsormidium reveal a land plant-like defense reaction. PLoS ONE.

[13] Lin C-S, Wu J-T (2014). Tolerance of soil algae and cyanobacteria to drought stress. J. Phycol..

[14] Ohtaka K, Hori K, Kanno Y, Seo M, Ohta H (2017). Primitive auxin response without TIR1 and Aux/IAA in the charophyte alga *Klebsormidium nitens*. Plant Physiol..

[15] Montero H (2021). A mycorrhiza-associated receptor-like kinase with an ancient origin in the green lineage. Proc. Natl. Acad. Sci..

[16] Stoyneva-Gärtner M (2019). Review on the biotechnological and nanotechnological potential of the streptophyte genus Klebsormidium with pilot data on its phycoprospecting and polyphasic identification in Bulgaria. Biotechnol. Biotechnol. Equip..

[17] Xu Z (2021). Assessment of a novel oleaginous filamentous microalga *Klebsormidium* sp. Lgx80 (Streptophyta, Klebsormidiales) for biomass and lipid production1. J. Phycol..

[18] Alves GSC (2017). Differential fine-tuning of gene expression regulation in coffee leaves by CcDREB1D promoter haplotypes under water deficit. J. Exp. Bot..

[19] Kim S (2021). Development of a versatile copper-responsive gene expression system in the plant-pathogenic fungus *Fusarium graminearum*. Mol. Plant Pathol..

[20] Windhagauer M (2021). Characterisation of novel regulatory sequences compatible with modular assembly in the diatom *Phaeodactylum tricornutum*. Algal Res..

[21] Bustin SA (2009). The MIQE guidelines: Minimum information for publication of quantitative real-time PCR experiments. Clin. Chem..

[22] Dong M (2012). The validity of a reference gene is highly dependent on the experimental conditions in green alga *Ulva linza*. Curr. Genet..

[23] Wang M, Wang Q, Zhang B (2013). Evaluation and selection of reliable reference genes for gene expression under abiotic stress in cotton (*Gossypium hirsutum* L.). Gene.

[24] Tang X, Zhang N, Si H, Calderón-Urrea A (2017). Selection and validation of reference genes for RT-qPCR analysis in potato under abiotic stress. Plant Methods.

[25] Lee M-A, Guo R, Ebenezer V, Ki J-S (2015). Evaluation and selection of reference genes for ecotoxicogenomic study of the green alga *Closterium ehrenbergii* using quantitative real-time PCR. Ecotoxicology.

[26] Mou S (2015). Reference genes for gene expression normalization in *Chlamydomonas* sp ICE-L. by quantitative real-time RT-PCR. J. Plant Biochem. Biotechnol..

[27] Fan J, Xu H, Li Y (2016). Transcriptome-based global analysis of gene expression in response to carbon dioxide deprivation in the green algae *Chlorella pyrenoidosa*. Algal Res..

[28] Monte I (2020). An ancient COI1-independent function for reactive electrophilic oxylipins in thermotolerance. Curr. Biol..

[29] Chatelain P, Astier J, Wendehenne D, Rosnoblet C, Jeandroz S (2021). Identification of partner proteins of the algae *Klebsormidium nitens* NO synthases: Toward a better understanding of NO signaling in eukaryotic photosynthetic organisms. Front. Plant Sci..

[30] Serrano-Pérez E (2022). Transcriptomic and metabolomic response to high light in the charophyte alga *Klebsormidium nitens*. Front. Plant Sci..

[31] Simon DF, Descombes P, Zerges W, Wilkinson KJ (2008). Global expression profiling of *Chlamydomonas reinhardtii* exposed to trace levels of free cadmium. Environ. Toxicol. Chem..

[32] Shi J (2012). Reference gene selection for qPCR in *Ammopiptanthus mongolicus* under abiotic stresses and expression analysis of seven ROS-scavenging enzyme genes. Plant Cell Rep..

[33] Moraes GP (2015). Evaluation of reference genes for RT-qPCR studies in the leaves of rice seedlings under salt stress. Genet. Mol. Res..

[34] Pfaffl MW, Tichopad A, Prgomet C, Neuvians TP (2004). Determination of stable housekeeping genes, differentially regulated target genes and sample integrity: BestKeeper—Excel-based tool using pair-wise correlations. Biotechnol. Lett..

[35] Vandesompele J (2002). Accurate normalization of real-time quantitative RT-PCR data by geometric averaging of multiple internal control genes. Genome Biol..

[36] Silver N, Best S, Jiang J, Thein SL (2006). Selection of housekeeping genes for gene expression studies in human reticulocytes using real-time PCR. BMC Mol. Biol..

[37] Andersen CL, Jensen JL, Ørntoft TF (2004). Normalization of real-time quantitative reverse transcription-PCR data: A model-based variance estimation approach to identify genes suited for normalization, applied to bladder and colon cancer data sets. Cancer Res..

[38] Pihur V, Datta S, Datta S (2009). RankAggreg, an R package for weighted rank aggregation. BMC Bioinform.

[39] Ichimura, T. Sexual cell division and conjugation-papilla formation in sexual reproduction of Closterium strigosum. In *Int. Symp. Seaweed Res.* (1971).

[40] Oñate-Sánchez L, Vicente-Carbajosa J (2008). DNA-free RNA isolation protocols for *Arabidopsis thaliana*, including seeds and siliques. BMC Res. Notes.

[41] Ramakers C, Ruijter JM, Deprez RHL, Moorman AFM (2003). Assumption-free analysis of quantitative real-time polymerase chain reaction (PCR) data. Neurosci. Lett..

[42] Ganger MT, Dietz GD, Ewing SJ (2017). A common base method for analysis of qPCR data and the application of simple blocking in qPCR experiments. BMC Bioinform..

[43] Li L, Li N, Fang H, Qi X, Zhou Y (2020). Selection and validation of reference genes for normalisation of gene expression in *Glehnia littoralis*. Sci. Rep..

[44] Zhang B-B (2021). Reference gene selection for expression studies in the reproductive axis tissues of Magang geese at different reproductive stages under light treatment. Sci. Rep..

[45] Huggett J, Dheda K, Bustin S, Zumla A (2005). Real-time RT-PCR normalisation; strategies and considerations. Genes Immun..

[46] Borges A, Tsai SM, Caldas DGG (2012). Validation of reference genes for RT-qPCR normalization in common bean during biotic and abiotic stresses. Plant Cell Rep..

[47] Czechowski T, Stitt M, Altmann T, Udvardi MK, Scheible W-R (2005). Genome-wide identification and testing of superior reference genes for transcript normalization in Arabidopsis. Plant Physiol..

[48] Løvdal T, Lillo C (2009). Reference gene selection for quantitative real-time PCR normalization in tomato subjected to nitrogen, cold, and light stress. Anal. Biochem..

[49] Eprintsev AT, Fedorin DN, Dobychina MA, Igamberdiev AU (2018). Regulation of expression of the mitochondrial and peroxisomal forms of citrate synthase in maize during germination and in response to light. Plant Sci..

[50] Gai Z (2020). Exogenous abscisic acid induces the lipid and flavonoid metabolism of tea plants under drought stress. Sci. Rep..

[51] Li Y (2020). Selection of reference genes for qRT-PCR analysis in medicinal plant glycyrrhiza under abiotic stresses and hormonal treatments. Plants.

[52] Zhou D (2020). OsPLS4 is involved in cuticular wax biosynthesis and affects leaf senescence in rice. Front. Plant Sci..

[53] Hao Z, Wei M, Gong S, Zhao D, Tao J (2016). Transcriptome and digital gene expression analysis of herbaceous peony (*Paeonia lactiflora* Pall.) to screen thermo-tolerant related differently expressed genes. Genes Genomics.

[54] Chen K (2020). BONZAI proteins control global osmotic stress responses in plants. Curr. Biol..

[55] Zeba N (2009). Heat-inducible C3HC4 type RING zinc finger protein gene from *Capsicum annuum* enhances growth of transgenic tobacco. Planta.

[56] Agarwal P, Khurana P (2018). Characterization of a novel zinc finger transcription factor (TaZnF) from wheat conferring heat stress tolerance in Arabidopsis. Cell Stress Chaperones.

[57] Kim D-Y, Jin J-Y, Alejandro S, Martinoia E, Lee Y (2010). Overexpression of AtABCG36 improves drought and salt stress resistance in Arabidopsis. Physiol. Plant..

[58] Saha J, Sengupta A, Gupta K, Gupta B (2015). Molecular phylogenetic study and expression analysis of ATP-binding cassette transporter gene family in *Oryza sativa* in response to salt stress. Comput. Biol. Chem..

[59] McNeilly D, Schofield A, Stone SL (2018). Degradation of the stress-responsive enzyme formate dehydrogenase by the RING-type E3 ligase keep on going and the ubiquitin 26S proteasome system. Plant Mol. Biol..

[60] Gupta D, Tuteja N (2011). Chaperones and foldases in endoplasmic reticulum stress signaling in plants. Plant Signal. Behav..

[61] Kayum MdA (2017). Genome-wide characterization and expression profiling of PDI family gene reveals function as abiotic and biotic stress tolerance in Chinese cabbage (*Brassica rapa* ssp. pekinensis). BMC Genomics.

[62] Yan C, Duan W, Lyu S, Li Y, Hou X (2017). Genome-wide identification, evolution, and expression analysis of the ATP-binding cassette transporter gene family in *Brassica rapa*. Front. Plant Sci..

[63] Ma R, Xu S, Zhao Y, Xia B, Wang R (2016). Selection and validation of appropriate reference genes for quantitative real-time PCR analysis of gene expression in *Lycoris aurea*. Front. Plant Sci..

[64] Xie L (2019). Selection of reference genes for real-time quantitative PCR normalization in the process of *Gaeumannomyces graminis* var. tritici infecting wheat. Plant Pathol. J..

