# Supplementary material for: Reliable reference genes and abiotic stress marker genes in Klebsormidium nitens
Source: Sci Rep. 2022 Nov 8;12:18988. doi: 10.1038/s41598-022-23783-9 (PMC9643330; doi:10.1038/s41598-022-23783-9)

Supplementary figure S1. Specificity of RT-qPCR. **a.** Amplification of one specific amplicon for each candidate reference gene after RT-qPCR by electrophoresis on 2% agarose gel. M represent 1 kpb Plus DNA ladder (ThermoFischer). **b.** Melting curves of each candidate reference genes.

a.

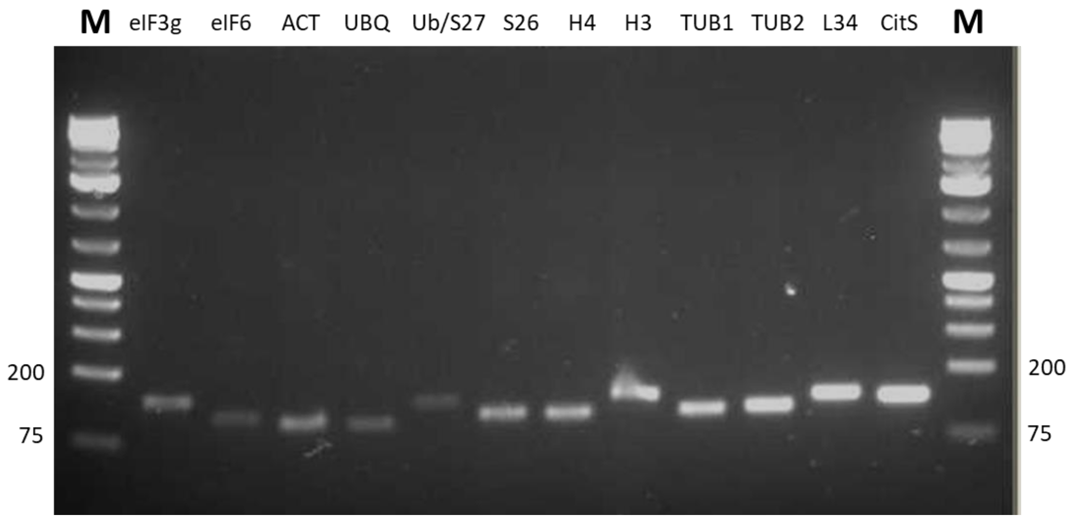

b.

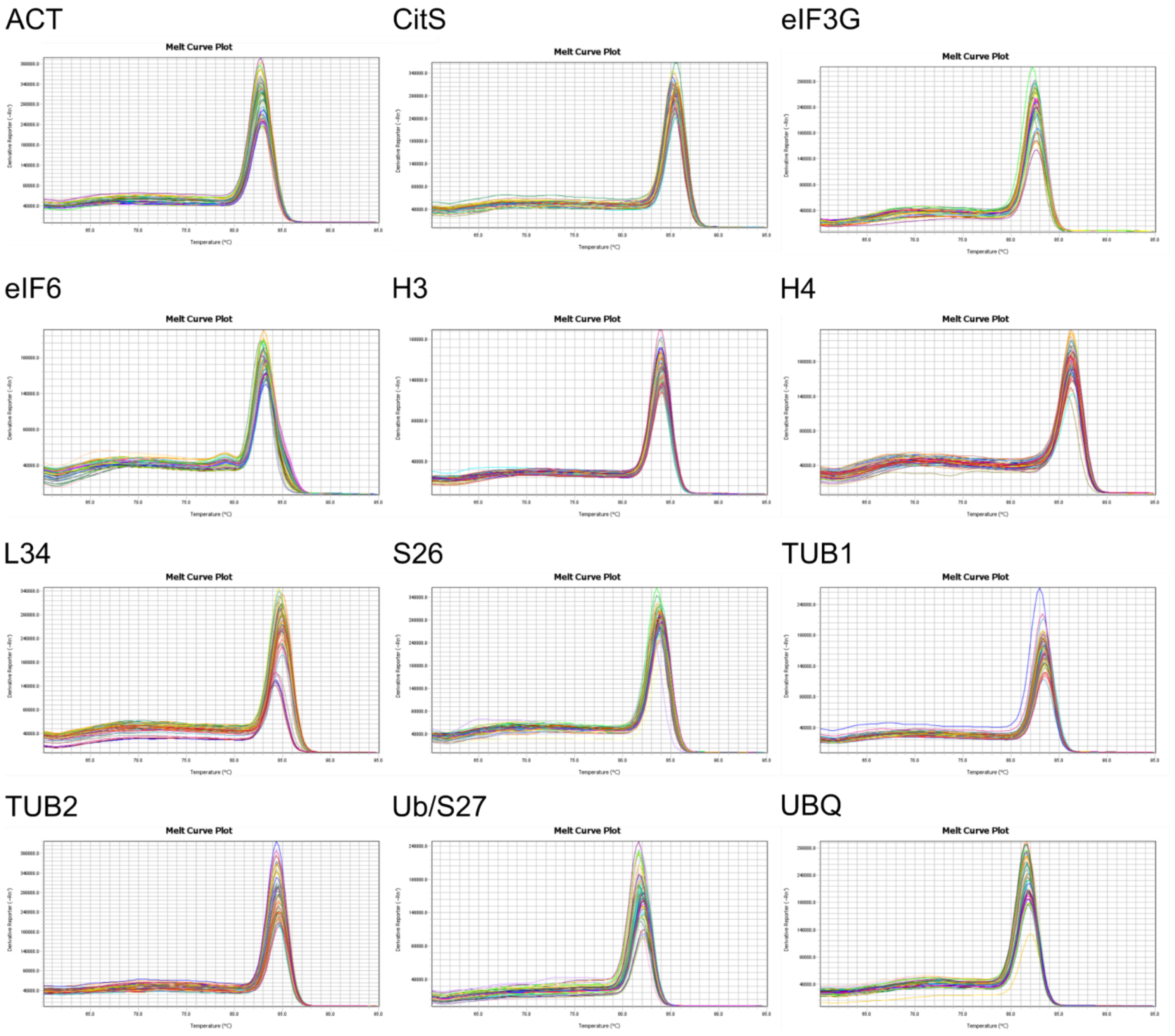

Supplement: Supplementary file 1 — Supplementary Figure S1. [file 41598_2022_23783_MOESM1_ESM.pdf]
