# Supplementary material for: Reliable reference genes and abiotic stress marker genes in Klebsormidium nitens
Source: Sci Rep. 2022 Nov 8;12:18988. doi: 10.1038/s41598-022-23783-9 (PMC9643330; doi:10.1038/s41598-022-23783-9)
Supplement: Supplementary file 2 — Supplementary Table S1. [file 41598_2022_23783_MOESM2_ESM.docx]

**Table S1.** Stress marker genes and associated primer set used in RT-qPCR in *K. nitens*. The tag locus of homologous genes on *A. thaliana* was referenced if applicated. Amplicon size and RT-qPCR efficiency (E) have been indicated. List of stress conditions tested for each gene were listed in the last column. HS: heat shock, SS: salt stress, PID: PEG Induced Dehydration.

| **Gene name** | **Gene symbol** | **Gene ID** | **Locus tag of *A. thaliana* homolog** | **Primer sequence (5’-3’) forward/reverse** | **Size (bp)** | **E** | **Stress** |
| --- | --- | --- | --- | --- | --- | --- | --- |
| ATP-binding cassette transporter | *ABC* | kfl00616_0050 | - | GCGTGGTATCTTGACCCTGT/  TCAGCCACGCTAACAATCTG | 116 | 1.902 | HS, SS, PID |
| Autophagy protein 5 | *APG5* | kfl00281_0120 | AT5G17290 | TGGCAGTTGACGGTTCATT/  GCCTCTTTCAGAGCGTTCAT | 101 | 1.856 | HS, SS, PID |
| Chloroplast heat shock protein hsp70-1 | *BIP5* | kfl00460_0030 | AT5G42020 | GCATACACGAAGAATGGAGACA/  CCGCCATCTTCCTACCAATAAA | 115 | 1.887 | HS, SS, PID |
| Heat shock protein (hsp 70) family | *BIP6* | kfl00190_0180 | AT5G42020 | GACCTGCTTCTGCTGGATATT/  CTCTCCCTGGAGGACCTTAAT | 117 | 1.866 | HS, SS, PID |
| Calcium-dependent phospholipid-binding Copine family protein | *BONZAI* | kfl00719_0030 | AT5G61900 | GTGACCCATTTCTCTTCCTCTC/  AGCGTGTTGTGCTTCACT | 85 | 1.894 | HS, SS, PID |
| Pti1 kinase-like protein | *CARK1* | kfl00169_0040 | AT3G17410 | TCACCTTGGCAGAGTTGAAG/  CGTCTTGCATCGTTCCGTAATA | 102 | 1.888 | HS, SS, PID |
| Cytochrome P450 | *cP450* | kfl00038_0230 | AT2G40890 | CGTTTAGGGACAGGGAGCTG/  CAGGTTCTTGAGCGTCAGGT | 143 | 1.898 | PID |
| E2.3.1.57, speG ; diamine N-acetyltransferase | *dANAT* | kfl00222_0190 | AT2G39030 | GGAGACGGACGATCACAAGG/  ACTCGGGCAACACAAAGAGA | 110 | 1.898 | PID |
| DnaJ heat shock family protein | *ERDJ* | kfl00061_0220 | AT3G62600 | TGCAGGGCGGAATTACTATG/  TCTTCGGTTGGGTTCTTGTC | 120 | 1.888 | HS, SS, PID |
| Formate dehydrogenase | *FDH* | kfl00145_0170 | - | GGACCTTGACACCTTCCTCA/  GCCCTTCTTCATCTTGGACA | 112 | 1.894 | HS, SS, PID |
| Glutathione S-transferase family protein | *GST* | kfl00151_0280 | - | CCCACCTTGCCTACACAGAT/  AGTACATGGCGGACACCTTC | 135 | 1.880 | HS, SS, PID |
| Heat shock protein | *HSP* | kfl00404_0080 | AT2G19310 | TCTCTGACTCCAGCTCAGGG/  AGGTCCGCCTCAAAAACGTA | 116 | 1.781 | HS |
| Pyr-redox 2 domain containing protein | *Hypo20* | kfl00020_0050 | - | GATTCCGTTCGACTTCCTGA/  TTGATCCCCTCGTATTGCTC | 108 | 1.830 | HS, SS, PID |
| Hypothetical protein | *Hypo272* | kfl00272_0070 | AT5G39520 | CGTCGCGTCTAAAGTGGCTA/  CTCAAGCGGAACCCCATACA | 112 | 1.901 | PID |
| Hypothetical protein | *Hypo434* | kfl00434_0040 | - | GGATACCGCGGACTACAAGA/  ATTGAGTGTCCCTTGCTTGG | 103 | 1.851 | HS, SS, PID |
| Hypothetical protein | *Hypo44* | kfl00044_0060 | AT5G66090 | CAGCAGCTAATTGGTCGCAC/  CTCGCTGGGGATCTCTGTTG | 141 | 1.871 | HS |
| Mitochondrial transcription termination factor | *mTERF* | kfl00643_0060 | AT1G78930 | CGCCCCAATGTGGAATACCT/  TCGGCACAAACTTCTCCTCC | 115 | 1.874 | HS |
| NAD(P)-binding Rossmann-fold superfamily protein | *NbRf* | kfl00021_0170 | - | GAAGCTGAACGTGCTCATCA/  AGCTGGAAGAGCAGGAAGTG | 120 | 1.891 | HS, SS, PID |
| 3-oxoacyl-(acyl-carrier-protein) reductase | *OAR* | kfl00896_0010 | - | CTCCAAAGGCAGGGACACTA/  GCCGTAGGTGGTGTCAAACT | 148 | 1.887 | HS, SS, PID |
| PDIA1, P4HB; protein disulfide-isomerase A1 | *PDI1* | kfl00120_0050 | AT5G60640 | TCTTTGGGTTCCTTCTCCTTTC/  GCACCTCATCATCTTCGTACTC | 102 | 1.767 | HS, SS, PID |
| Esterase/lipase/thioesterase family protein | *PES1* | kfl00048_0280 | AT1G54570 | GGCCTGGTGAGATTTGTGGA/  TACACGTCTGGGTTTCTGGC | 134 | 1.906 | PID |
| Peptidase S41 family protein | *S41* | kfl00140_0150 | AT3G57680 | CTGGGACTACTGTGCGGATC/  AACACGGGCGAGAAGACAAT | 103 | 1.905 | PID |
| SGT1B homolog | *SGT1B* | kfl00147_0020 | AT4G23570 | TGGACCCCAAAAACTCCCAG/  CGGAGACTCTTTCGCTGGTT | 149 | 1.888 | HS |
| Hypothetical protein | *TBF1* | kfl00260_0030 | AT4G36990 | CCTTGGCTTCTCTGGAAACA/  GTCGTCTGTGCTGACATCAT | 92 | 1.911 | HS, SS, PID |
| Vesicle-associated membrane protein | *Vamp* | kfl00515_0100 | AT4G15780 | TTGGTCGTCGCAACAGAAGA/  CGCAGCTTAGGCCCAAAATC | 149 | 1.895 | HS |
| Zinc finger, RING-type domain containing protein | *ZnFR* | kfl00034_0310 | AT5G47610 | CGACGAGTGTCCTATCTGCC/  CGACCCACTCCGCTATACAC | 107 | 1.861 | HS |
